# Transwell Insert-Embedded Microfluidic Devices for Time-Lapse Monitoring of Alveolar Epithelium Barrier Function under Various Stimulations

**DOI:** 10.3390/mi12040406

**Published:** 2021-04-06

**Authors:** Shu-Han Chang, Ping-Liang Ko, Wei-Hao Liao, Chien-Chung Peng, Yi-Chung Tung

**Affiliations:** 1 Institute of Biophotonics, National Yang-Ming University, Taipei 11221, Taiwan; sheg65566@gmail.com; 2 Institute of Biophotonics, National Yang Ming Chiao Tung University, Taipei 11221, Taiwan; 3 Research Center for Applied Sciences, Academia Sinica, Taipei 11529, Taiwan; b97209014@gmail.com (P.-L.K.); jamliao@gate.sinica.edu.tw (W.-H.L.); vp@gate.sinica.edu.tw (C.-C.P.); 4 Department of Mechanical Engineering, National Taiwan University, Taipei 10617, Taiwan; 5 College of Engineering, Chang Gung University, Taoyuan 33302, Taiwan

**Keywords:** transwell insert, microfluidics, air-liquid interface (ALI) cell culture, trans-epithelial electrical resistance (TEER), alveolar epithelium, atmospheric exposure

## Abstract

This paper reports a transwell insert-embedded microfluidic device capable of culturing cells at an air-liquid interface (ALI), mimicking the in vivo alveolar epithelium microenvironment. Integration of a commercially available transwell insert makes the device fabrication straightforward and eliminates the tedious device assembly processes. The transwell insert can later be detached from the device for high-resolution imaging of the cells. In the experiments, the cells showing type-I pneumocyte markers are exploited to construct an in vitro alveolar epithelium model, and four culture conditions including conventional liquid/liquid culture (LLC) and air–liquid interface (ALI) cell culture in normal growth medium, and ALI cell culture with inflammatory cytokine (TNF-α) stimulation and ethanol vapor exposure are applied to investigate their effects on the alveolar epithelium barrier function. The barrier permeability is time-lapse monitored using trans-epithelial electrical resistance (TEER) measurement and immunofluorescence staining of the tight junction protein (ZO-1). The results demonstrate the functionalities of the device, and further show the applications and advantages of the constructed in vitro cell models for the lung studies.

## 1. Introduction

The lung is one of the most important organs in a human body, and its key function is to perform oxygen transportation from the atmosphere into the bloodstream to oxygenate entire body through the circulatory system. Lungs can also help to release carbon dioxide from the bloodstream to the atmosphere [1]. The gas exchange takes place at blood-air barriers in alveoli, hollow cavities with thin-walled structures, located at the end of the respiratory tract. In an alveolus, the majority of surface area (>95%) is covered by large and thin type-I pneumocytes [2]. Since the lung directly faces ambient environments, exposure to hazardous atmospheres like air pollution and volatile organic compounds (VOCs) can cause damage to the pneumocytes [3,4,5,6,7]. In addition, pathological conditions such as chronic obstruction pulmonary disease (COPD) and, recently, COVID-19 pneumonia may also impair proper functions of the pneumocytes. The dysfunction of the pneumocytes can lead to impaired gas exchange, alveolar flooding and/or collapse, and systematic inflammatory response syndrome [8,9,10,11,12].

In order to study effects of atmospheric exposure and diseases on the primary lung functions, animal experiments are commonly exploited. However, due to complex biological compositions (over 40 cell types in a lung) and physical environments (e.g., substrate stretching and air/liquid interface), it is challenging to investigate the underlying mechanisms and pathways of lung dysfunction. In addition, without representative in vitro cell culture models to reconstitute in vivo microenvironments, it is infeasible to elucidate the effects of single factors on the pneumocytes [13]. As a result, it is highly desired to develop an in vitro cell model capable of recapitulating the physiological arrangement of the cells as well as performing time-lapse quantitative analysis on the cells under various atmospheric exposure and pathological conditions.

Microfluidic cell culture devices have recently obtained, increasing attention due to its desired advantages, including—capability of controlling culture microenvironments in spatial and temporal domains, and mimicking physiological arrangements of cells in vivo [14,15,16,17,18]. Among various applications, the devices have been demonstrated to provide insightful information and fundamental understanding for lung studies. For example, Huh et al. developed a microfabricated airway system integrated with computerized air–liquid two-phase microfluidics that enables on-chip engineering of human airway epithelia, and precise reproduction of physiologic or pathologic liquid plug flows found in the respiratory system [19]. In addition, a biomimetic microfluidic device is developed to reconstitute the critical functional alveolar–capillary interface of the human lung. The bioinspired microdevice reproduces complex integrated organ-level responses to bacteria and inflammatory cytokines introduced into the alveolar space [20]. To investigate how the combination of fluid and solid stresses commonly found in alveoli affects the alveolar epithelial cells, a novel multiple-layer microfluidic device simulating the in vivo physical microenvironments was also constructed in the previous study [21]. Furthermore, a biomimetic microfluidic device capable of reconstituting physiological physical microenvironments in the lungs during fetal development for cell culture is developed. The device can be exploited to investigate effects of drug treatment and physical stimulation on surfactant protein expression of lung epithelial cells [22].

Despite various microfluidic cell culture devices mimicking the cell arrangement in lungs, they suffer several disadvantages retarding their practical usage, especially for atmospheric exposure study. Most of the existing devices are constructed with multiple layers and complicated structures requiring sophisticated fabrication processes. The multiple layer architecture limits the imaging capability due to the requirement of long working distance objectives. Moreover, the devices are not optimized for introduction of vapor contents to conduct atmospheric exposure experiments mimicking stimulation during respiration. The slender microfluidic channel geometry limits the passive diffusion efficiency for the vapor samples in contact with the cultured epithelial cells. As a result, active actuation pumping may be required to deliver atmospheric samples for the experiments, which makes experimental setup complicated for practical usage in biological labs and scaling up of the device for multiple tests challenging.

In order to construct an in vitro alveolar epithelium model culturing cells at an air–liquid interface (ALI), porous membranes need to be integrated within the devices [23,24]. The integration often requires additional etching or lamination process lowering the fabrication yield. In addition, without capability of performing physiological functional assays, the devices are only capable of providing limited information based on conventional biochemistry analysis that cannot be easily compared with those obtained from animal experiments and further translated to clinical observation. The commercially available transwell design allows the cell culture at air-liquid interface; however, it is not suitable for the vapor exposure experiments. The conventional transwell inserts are designed with through holes on the edges of the inserts to prevent the air squeezing between the inserts and the wells. Therefore, the solution in the well underneath the transwell insert is directly exposed to the atmospheric condition, which leads to direct dissolution of the vapor into the solution. As a result, the effects of the vapor exposure on the cell layers cannot be accurately characterized. Consequently, a transwell insert-embedded microfluidic cell culture device is developed in this paper to perform ALI cell culture for an in vitro alveolar epithelium model. The device takes advantage of a commercially available transwell insert with a ready-to-use porous membrane for ALI cell culture to eliminate the tedious fabrication process. Moreover, the transwell insert can be later detached for further staining and imaging to investigate the cellular responses after the experiments.

To ensure the functionalities of the device for vapor exposure on the ALI cell culture, an alveolar epithelial cell line with type-I pneumocyte-like properties is exploited to be cultured in the device in the experiments. In order to compare the effects of culture formats, conventional liquid/liquid culture (LLC) and ALI culture are performed. In addition, an inflammatory cytokine and ethanol vapor are also used to stimulate the cells cultured at the ALI to mimic a pathological epithelium and an epithelium exposed to hazardous VOC atmosphere, respectively. During the experiments, time-lapse trans-epithelial electrical resistance (TEER) measurements across the formed alveolar epithelium cultured under different conditions are performed. TEER measurement offers a quantitative approach to measure the tight junction expression between cells that plays an important role in solute transportation through the paracellular space [23,25,26]. The impedance values can be measured in real time without damaging the cells; therefore, it is suitable for time-lapse monitoring during the culture. In addition, immunofluorescence staining is also conducted to observe the cellular response in terms of junction protein expression. The results confirm the functionalities of the developed device, and show the time-course variation of tight junction expression of the alveolar epithelia under various conditions. Integrating conventional transwell inserts with the microfluidic channels, the developed device can be further applied to construct in vitro blood–air barrier models with multiple cell types co-cultured under dynamic flow culture conditions for research in respiratory systems.

## 2. Materials and Methods

### 2.1. Design and Fabrication of the Microfluidic Device

**Figure 1:**
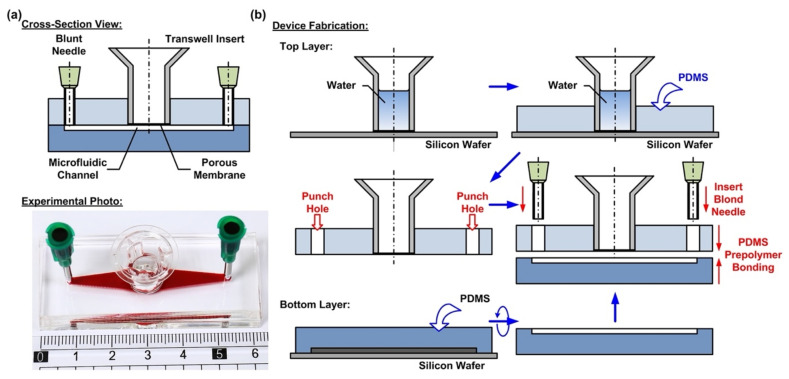
(**a**) Cross-sectional view and the experimental photo of the transwell insert-embedded microfluidic device capable of culturing cells at the air–liquid interface (ALI), mimicking alveolar epithelium. (**b**) Fabrication process of the transwell insert-embedded microfluidic device based on the soft lithography method.

In order to culture the cells at the air–liquid interface (ALI) and investigate the responses of the cells exposed to different atmospheric environments, a microfluidic cell culture device with an embedded commonly used transwell insert is developed in this paper. The microfluidic device is made of an elastomeric material, polydimethylsiloxane (PDMS), due to its cell culture compatibility, optical transparency, and gas permeability [27,28,29]. The device consists of two PDMS layers—a top layer with an embedded transwell insert, and a bottom layer with a microfluidic channel pattern, as shown in Figure 1a. The integration of the commonly used commercially available transwell insert provides a simple and straightforward solution to culture cells on a porous membrane for the ALI culture. In addition, the transwell insert can be easily detached from the device later for high-resolution imaging and further analysis.

The fabrication process of the device is based on the well-developed soft lithography process, as shown in Figure 1b. First, PDMS prepolymer (Sylgard 184, Dow Corning Co., Midland, MI, USA) is prepared by mixing base and curing agent at a ratio of 10:1 (*w*/*w*). The top layer is made by carefully pouring the PDMS prepolymer on a silicon wafer on which a transwell insert with 0.4 µm pore (3470, Corning Costar, Corning, NY, USA) is temporarily attached. The wafer is silanized with 1H,1H,2H,2H-perfluorooctyltrichlorosilane (78560-45-9, Alfa Aesar, Ward Hill, MA, USA) in a desiccator for more than 30 min at room temperature to prevent undesired bonding of PDMS to the wafer before the process. In order to prevent the prepolymer flowing into the transwell insert to block the porous membrane, deionized water is filled into the transwell insert before pouring the prepolymer. The bottom PDMS layer is fabricated using the replica molding process. For the proof-of-concept experiments, the microfluidic channel is designed with a width, length, and height of 3, 30, and 1 mm, respectively. The channel dimensions can be further adjusted as microfluidic channels to simulate in vivo physical and pathological microenvironments under dynamic flow conditions. In the process, a laser-cut 1-mm thick acrylic layer with channel geometries attached onto a silicon wafer using a double-side tape is exploited as a mold, and the mold is also silanized before the process. The PDMS layers are cured at 60 °C for more than 4 h in an oven. After curing, the PDMS layers are then peeled from the silicon wafers, and the inlet and outlet of the microfluidic channel are punched using a biopsy punch with a diameter of 2 mm on the top layer.

To assemble the device, a thin layer of PDMS prepolymer, prepared by mixing base and curing agent with a ratio of 3:2 (*w*/*w*) is first spun on a silanized silicon wafer as an adhesive layer. The bottom PDMS layer is then placed on the adhesive layer with the channel pattern surface facing down to coat the prepolymer on the surface. The two layers are then aligned and bonded with each other by placing the assembled device in a 60 °C oven for more than 16 h. The thin PDMS adhesive layer provides reliable leakage-free bonding between the layers and does not block either the porous membrane or the microfluidic channel. Figure 1a shows the experimental photo of the fabricated device.

### 2.2. Cell Culture

In this paper, a human lung alveolar epithelial cell line is exploited to investigate the cellular responses in alveoli of a lung under various cytokine and atmospheric exposure stimulations. In order to better simulate a normal alveolar epithelium, commercially available alveolar epithelial cells (Cl-hAELVi, InSCREENeX GmbH, Braunschweig, Germany) with type-I pneumocyte-like properties (e.g., expression of Caveolin-1 and absence of Surfactant Protein C, low permeability, and capability of being cultured at an air–liquid interface) were used [23,30]. With the normal cells derived from humans, the model is anticipated to provide better predictive capability to be translated to clinical observation and therapy.

**Figure 2:**
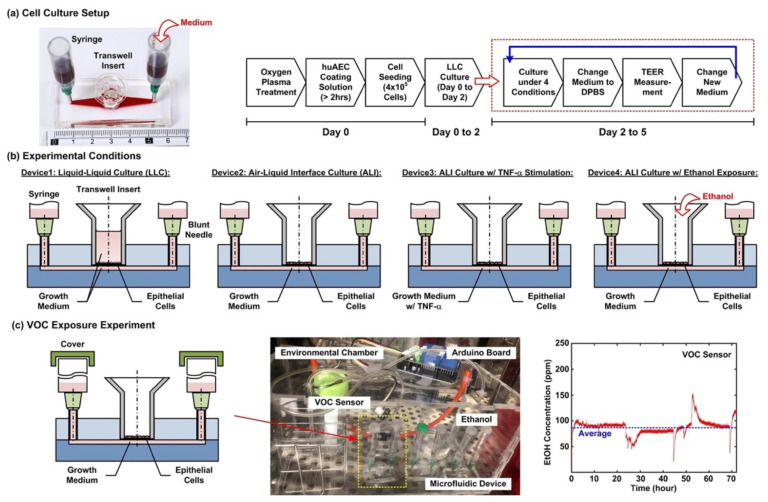
(**a**) Experimental photo of the cell culture setup, and an illustration of the cell culture experimental flow in the microfluidic device. (**b**) Four cell culture conditions tested in the experiments to study time-lapse trans-epithelial electrical resistance (TEER) variation of the epithelium cultured in different formats and under the cytokine and VOC exposure stimulations. (**c**) Schematic and photo of the experimental setup to perform the VOC (ethanol) exposure experiments on the developed transwell-embedded microfluidic device in a cell incubator.

The stocks of hAELVi cells are cultured in huAEC medium (INS-ME-1013, InSCREENeX GmbH) according to the protocol provided by the supplier. Figure 2a shows the cell culture setup and experimental flow conducted in the experiments. For the cell culture in the microfluidic devices, the device is first treated with oxygen plasma to make the microfluidic channel walls hydrophilic. Dulbecco’s phosphate-buffered saline (DPBS) (Gibco 14190, ThermoFisher Scientific, Waltham, MA, USA) solution is then filled into the microfluidic channel from the inlet, and 200 µL of huAEC coating solution (INS-SU-1018, InSCREENeX GmbH) is pipetted into the transwell insert. After more than a 2 hour-incubation, two blunt needles are then inserted into the inlet and outlet holes as interconnections between microfluidic channel and syringes as medium reservoirs for the following cell culture experiments. The DPBS and the coating solutions are replaced by the growth medium after the incubation. For cell seeding, the cells are dissociated using 0.25% trypsin-EDTA (Gibco 25200, Thermo Fisher Scientific). The cell suspensions for the experiments are made by centrifugation of the dissociated cells at 1000 rpm for 5 min at room temperature. Cell suspension in the growth medium with volume of 200 µL containing 4 × 10^5^ cells is pipetted into the transwell insert for the experiments. The cells are cultured for 2 days (Day 0 to 2) in the liquid environment with the growth medium to form confluent epithelium before changed to different culture conditions, and the medium is exchanged every day during the experiments.

After a 2 day-formation of the epithelium, four cell culture conditions are performed for 3 days (Day 2 to 5) to investigate cellular responses under different culture formats, cytokine stimulation, and volatile organic compound (VOC) exposure in the cell experiments. The four conditions as shown in Figure 2b are—(1) Device1: conventional liquid/liquid culture (LLC) of the cell as control for the experiments. The microfluidic channel and the transwell insert are filled with the medium during the culture; (2) Device2: cell culture at an air–liquid interface (ALI) to simulate the physiological environment in a lung. During the culture, only the microfluidic channel is filled with the medium, and the transwell is left empty to make the cells directly exposed to atmosphere; (3) Device3: cell culture at the ALI with tumor necrosis factor (TNF-α) stimulation to simulate an inflamed lung. The microfluidic channel is filled with the medium containing 10 ng/ml TNF-α (H8916, Sigma-Aldrich, St. Louis, MO, USA) during the culture; and (4) Device4: cell culture at the ALI and alcohol (ethanol) vapor-filled atmospheric environment to simulate a lung with inhalation of VOC. In order to establish an atmosphere filled with ethanol vapor, an environmental chamber equipped with a VOC sensor is constructed for the experiments as shown in Figure 2c. A container filled with ethanol (95%) is exploited to generate the vapor in the environmental chamber, and a VOC sensor (Grove-HCHO sensor, Seeed Technology Co., Shenzhen, China) capable of measuring alcohol vapor concentration interfaced with a microcontroller board (Arduino Uno Rev 3, Arduino) is exploited to measure and record the vapor concentration. During the experiments, the inlet and the outlet of the microfluidic channel are covered to prevent the ethanol vapor from directly dissolving into the medium. All the microfluidic device cell culture experiments are performed in a humidified cell incubator with controlled atmospheric conditions (5% CO_2_) and temperature (37 °C).

### 2.3. Cell Viability Assay

In order to observe the cell compatibility of the device and cell viability under different culture conditions, fluorescence imaging-based cell viability assays are performed in the experiments. In the experiments, the live and dead cells cultured in the device are stained using calcein AM (4 mM) (Invitrogen C3100MP, Thermo Fisher Scientific, Waltham, MA, USA) and ethidium homodimer-2 (EthD-2) (2 mM) (Invitrogen E3599, Thermo Fisher Scientific, Waltham, MA, USA), respectively. The nuclei stain, NucBlue Hoechst 33342 (Invitrogen R37605, Thermo Fisher Scientific, Waltham, MA, USA), is also used for cell counting and analysis. The stained cells are then imaged using an inverted fluorescence microscope (DMi8, Leica Microsystems, Wetzlar, Germany).

### 2.4. Trans-Epithelial Electrical Resistance (TEER) Measurement

**Figure 3:**
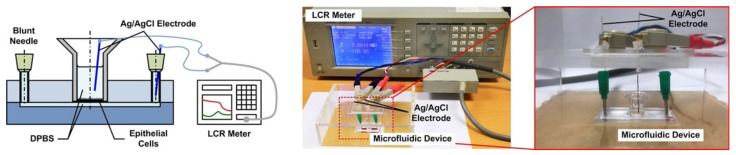
Schematic and experimental photo of the TEER measurement setup used in the experiments.

Transmembrane electrical resistance offers a quantitative approach to real-time measure the integrity of the tight junctions that govern solute transport across the paracellular space of live endothelial and/or epithelial monolayers. As a result, trans-epithelial electrical resistance (TEER) measurement is exploited to investigate the barrier function of the epithelium formed in the devices under various conditions in a time-lapsed manner. Figure 3 illustrates the experimental setup for the TEER measurement in the experiments. First, the device is taken out from the incubator, and the medium in the transwell insert (for the LLC experiments) and microfluidic channel are filled or replaced by the DPBS, and the entire device is incubated under room temperature for 10 min to reach equilibrium temperature. For the measurement, two Ag/AgCl electrodes (EP05, World Precision Instruments, Sarasota, FL, USA) with diameters of 0.5 mm are vertically inserted into the transwell insert and inlet or outlet of the microfluidic channel, respectively. An electrical LCR meter (WK6105, Wayne Kerr, New Taipei City, Taiwan) is then connected to the electrodes to measure the electrical impedance from 20 Hz to 1 MHz to ensure that the impedance spectrum agrees with the commonly adopted equivalent electrical circuit model for the cell layers [25]. The impedance measured at 20 Hz is used to approximate the DC resistance value for the TEER value calculation. The DPBS is removed or replaced by the medium in the device to restore its original experimental conditions, and the device is then put back into the incubator after the TEER measurement. The TEER measurements are performed daily from Day 2 to 5. The cells are dissociated from the membrane using the trypsin-EDTA after the measurement on Day 5, and the TEER measurement is then performed on the exact device without the cells cultured on top of the membrane as the blank value. The TEER value of the cell layer is then calculated as:

TEER = (Resistance Value w/Cells − Resistance Value w/o Cells) × Cell Culture Area
(1)

The cell culture area is the area of the porous membrane in the transwell insert, which is 0.33 cm^2^ in the experiments. For each cell culture condition, three independent experiments are conducted for statistical analysis. Statistical analysis is performed by ANOVA followed by Holm–Sidak tests.

### 2.5. Immunofluorescence Staining

In order to compare the TEER measurement results and the tight junction formation between the hAELVi cells cultured under various conditions, immunofluorescence staining and imaging of the tight junction protein, zonula occludens-1 (ZO-1), are performed in the experiments at the same time points as the TEER measurements. The cells are first washed using DPBS twice, and then fixed using 4% paraformaldehyde (PFA) for 15 min. After fixation, the cells are washed using DPBS three times. The cells are then treated with a blocking buffer composed of 0.5% bovine serum albumin (BSA) and 0.025% saponin in DPBS for one hour. The cells are then incubated with a ZO-1 antibody (21773-1-AP, Proteintech Group, Inc., Rosemont, IL, USA) in the blocking buffer (1:100) at 4 °C overnight. The cells are stained with CF 543 (20308, Biotium, Inc., Fremont, CA, USA) in the blocking buffer (1:400) for an hour. The nuclei of the cells are also stained using DAPI (4′, 6-diamidino-2-phenylindole) in DPBS. The stained cells are imaged using a confocal microscope (TCS SP5, Leica Microsystems, Wetzlar, Germany) in the experiments.

## 3. Results and Discussion

### 3.1. Cell Viability

**Figure 4:**
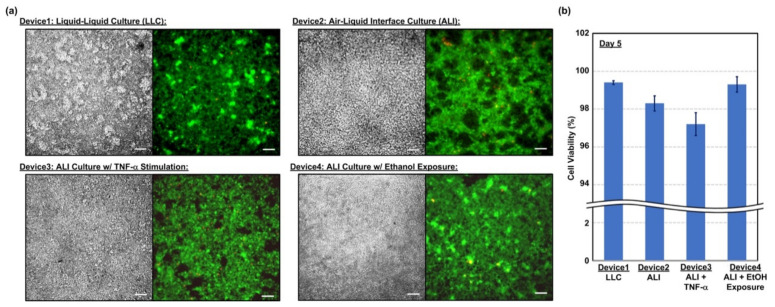
(**a**) Brightfield phase images and the fluorescence images of the hAELVi cells cultured in the devices for 5 days and under different conditions from Day 2 to 5. Scale bar is 20 µm. (**b**) The quantitative analysis results of the cell viability assay at Day 5.

The viability of the cells cultured in the microfluidic devices are characterized at Day 5 to confirm the cell compatibility of the device and investigate the effects of the culture conditions on the cell viability. Figure 4a shows the brightfield phase and fluorescence images of the cells cultured under various conditions and stained with calcein AM (green) and EthD-2 (red) for live and dead cells, respectively. The images show that the cells can well attach on the porous membranes of the transwell inserts for all four conditions, and most of the cells show green fluorescence, indicating their live status. The fluorescence images are further analyzed to quantify the viability, and the results are shown in Figure 4b. The plot shows that the cell viabilities for all four culture conditions are higher than 96% indicating the cell compatibility of the device and the cells can live well in the devices without significant damage. The viability of the cells cultured with TNF-α stimulation is slightly lower than those under other conditions. The lower viability may be caused by alveolar epithelial dysfunction triggered by the TNF-induced death signaling [31].

### 3.2. TEER Measurement

**Figure 5:**
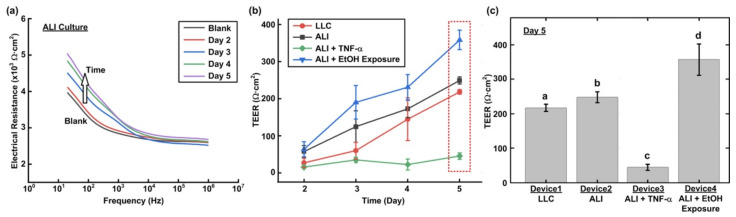
(**a**) Electrical impedance spectrum measured on the epithelium formed in the device at the ALI using the LCR meter in the experiments from Day 2 to 5 and the device after removing the cells by trypsinization at Day 5 (Blank). (**b**) The TEER values calculated from the impedance spectrum measurement results obtained in the cell culture experiments under different conditions from Day 2 to 5 (n = 3). (**c**) Comparison of the TEER values measured on the epithelium cultured under difference conditions at Day 5 (a, b, c, d = *p* < 0.05, n = 3).

The TEER measurements are performed after the two-day cell culture in the devices. Figure 5a shows typical measured impedance spectrum curves directly obtained from the LCR meter. The spectrum curves are similar to those obtained in previous research, suggesting the feasibility of accurate TEER measurement in the transwell insert-embedded microfluidic device with the experimental setup [25]. The impedance curves measured with the cell layers are all above that measured on the same blank device after the cell dissociation. Furthermore, the measured spectrum curves move upward with greater values, especially in the lower frequency range, showing the increase of the resistance resulted from the tight junction formation of the cell layers through the culture period. The cell layer impedance spectrum can then be calculated by subtracting the spectrum curve obtained from the blank device from that obtained from the device with cells cultured on top of the porous membrane. The TEER value can then be approximated using the impedance value at the lowest measurement frequency (20 Hz).

In order to compare the TEER value variation of the cell layers cultured under different conditions, the calculated TEER values measured from Day 2 to 5 are plotted in Figure 5b. The plot shows that when the cells cultured in the normal growth medium without additional stimulations, the cells cultured at the ALI possess greater TEER values than those cultured in conventional liquid environment (LLC). The average TEER value of the cells cultured at the ALI reaches 248.9 (Ω·cm^2^) at Day 5 which is about 14.1% higher than that obtained from the LLC experiments, which is about 218.1 (Ω·cm^2^). When the cells are cultured at the ALI with the addition TNF-α stimulation from the basal side, the TEER value remains similar from Day 2 to 5 without significant increase (between 15.4 and 45.3 Ω·cm^2^) and becomes lower than that cultured in the growth medium without additional stimulations. The average value is 45.3 (Ω·cm^2^) at Day 5, which is about one fifth of the value obtained in the LLC experiments. The results suggest that the inflammatory cytokine, TNF-α, can alter tight junction protein expression and increase the junction permeability of the epithelium. As a result, it is confirmed that the experimental setup can be exploited to quantitatively characterize the barrier function based on the TEER measurement.

Interestingly, when the cells cultured at the ALI exposed to the ethanol vapor with an average concentration of 87.1 ppm (Figure 2c), the TEER value increases during the culture and reaches a value of 358.1 (Ω·cm^2^) at Day 5 that is even higher than that obtained from the experiments conducted using normal growth medium without additional stimulations (both LLC and ALI). The higher TEER values suggests that there is a possible increase of tight junction protein expression, which may lead to lower epithelium permeability, after the cells are exposed to the ethanol vapor. The results also confirm that the device can be exploited to study cellular responses of epithelium exposed to VOC vapor mimicking hazardous atmosphere conditions.

In order to statistically compare the epithelium formed in the device under different culture conditions after experiments, the TEER values obtained at Day 5 in the experiments are plotted, as shown in Figure 5c. At Day 5, the average TEER value obtained from the LLC control experiments in the Device1 is approximately 218.1 (Ω·cm^2^), and the value obtained from the ALI culture experiments without additional stimulations is significantly higher (14.1%) than that obtained from the control experiments. With the cytokine stimulation, the average TEER value of the TNF-α-stimulated cell layers cultured at the ALI is 45.3 (Ω·cm^2^), which is approximately 79.2% and 81.8% lower than those obtained in the LLC and ALI experiments performed with the growth medium, respectively. In contrast, the average TEER value of the cell layers cultured exposed to the ethanol vapor at the ALI is 358.1 (Ω·cm^2^), which is approximately 64.2% and 43.9% higher than those obtained in the LLC and ALI experiments performed with the growth medium, respectively.

### 3.3. Immunofluorescence Staining

**Figure 6:**
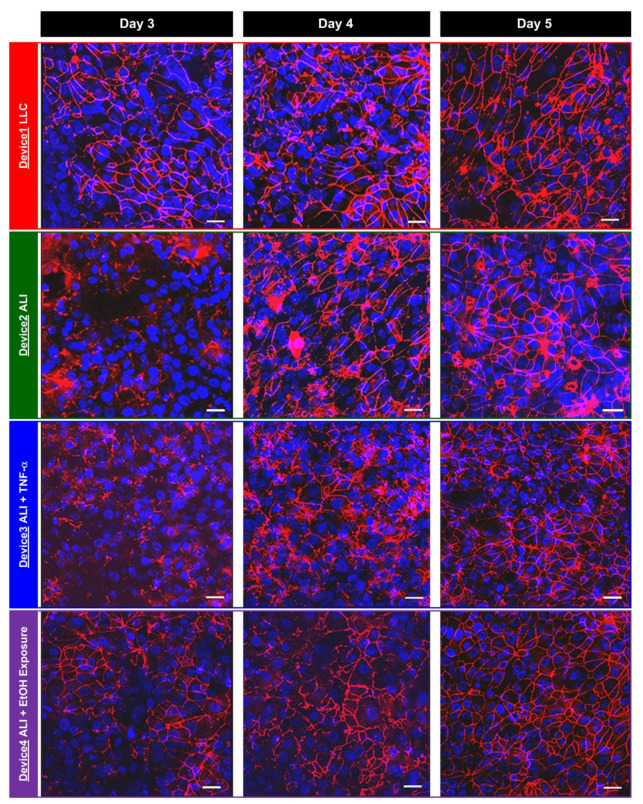
Fluorescence images of the epithelium formed by the hAELVi cells cultured in the devices under various conditions at Day 3, 4 and 5. The cells are stained with tight junction protein ZO-1 (red) and nuclei (blue). Scale bar is 20 µm.

For comparison, the immunofluorescence staining of the tight junction protein, ZO-1, is also performed on the hAELVi cells cultured under the different conditions at the same time points as the TEER measurements, and the captured confocal fluorescence images are shown in Figure 6. The images show that when the cells are cultured under LLC and ALI conditions without additional cytokine and VOC vapor stimulations, the tight junction protein levels increase during the culture. At Day 3, the ZO-1 expressions appear at the interfaces between some cells, and the expressions further increase at Day 4. At Day 5, most of the interfaces between the cells show ZO-1 expression, indicating the well-formed epithelium with tight junction protein expression between the cells can be obtained under both growth medium culture conditions. The results agree with the trends of the increasing TEER values and confirm that the epithelium can be successfully formed in the developed transwell insert-embedded microfluidic cell culture device.

For the cells cultured at the ALI, the tight junction protein (ZO-1) expression slightly increases from Day 3 to 4 when the cells are stimulated by the inflammatory cytokine TNF-α (Device 3) and remains similar from Day 4 to 5. In addition, the ZO-1 expression presents at only some of the cell interfaces, which is less than the expression observed in Device1 and Device2. In contrast, the tight junction protein expression keeps increasing from Day 3 to 5 when the cells are exposed to the ethanol vapor (Device 4), and presents at almost all the cell/cell interfaces. The immunofluorescence staining results also agree well with the TEER measurements on the cells under cytokine treatment and VOC exposure conditions, suggesting the impairing and enhancing roles of TNF-α and ethanol vapor on the tight junction formation of the alveolar epithelium, respectively.

The results show that when the cells are cultured in normal growth medium in different formats, the ALI culture can promote the epithelium tight junction formation for the specific hAELVi cells resulting in higher TEER value and ZO-1 expression [23]. With the inflammatory cytokine stimulation, the results agree with the previous observation suggesting that inflammatory processes, including secretion of TNF-α, can disrupt the barrier function and increase barrier permeability of epithelium found in both in vivo and in vitro models [31,32]. For the VOC exposure experiments, several studies have shown that alcohol ingestion can increase the alcohol vapor in the lung and further impair alveolar barrier function by increasing tight junction permeability [33,34]. However, we have not observed increased tight junction protein expression of alveolar epithelium cultured at the ALI when exposed to VOC (ethanol) vapor based on the in vitro models conducted in previous studies. Interestingly, inhalation of alcohol and certain volatile substances have been proposed for the treatment of acute pulmonary edema in early 1950s, and it is suggested that the vapor changes surface tension and decreases the amount of foaming in the respiratory passages [35,36]. The altered surface tension may also play an important role in the culture of the epithelium at the ALI and further increase the tight junction expression as shown in the experimental results. The detail underlying mechanism needs further advanced study, and the in vitro model developed in this paper can provide a powerful yet straightforward platform to investigate the effects of various vapor exposures on the epithelium functions in a time-lapsed manner.

## 4. Conclusions

In this paper, a transwell insert-embedded microfluidic cell culture device is developed to study alveolar epithelium barrier function based on an in vitro cell culture model. The transwell insert embedded architecture makes it feasible to culture the cells at the ALI for atmospheric environment exposure experiments. In addition, the device fabrication process is straightforward, without tedious assembly of multiple layers. Although the real-time imaging of the cells with high magnification ratio is infeasible due to the height of the bottom microfluidic channel and limited transparency of the membrane, the insert can be later detached from the device at the end point of the experiments for high resolution imaging without considering limited working distance of the objective. It is noted that the transwell insert cannot be reassembled to the microfluidic device due to possible leakage and additional undesired force application on the cells cultured on the porous membrane. The device is exploited to culture commercially available human alveolar epithelial cells to investigate epithelium barrier function under various culture conditions based on TEER measurement and immunofluorescence staining in a time-lapse manner. In the experiments, conventional LLC and ALI cell cultures are performed to compare the effects of culture format on the barrier function. The results show that the ALI culture can promote tight junction protein expression and reduce the barrier permeability. In addition, two types of stimulations—inflammatory cytokine and ethanol vapor exposure—are conducted on the cell culture at the ALI, and the results show the impairing and promotion of the barrier permeability by the cytokine stimulation and ethanol vapor exposure, respectively. The TEER measurement provides time-lapse observation capability on the same batch of cells. Consequently, the developed device can provide a practical platform to construct in vitro models for barrier function study under various physiological and pathological conditions. In addition, alveolar epithelial and endothelial cells can be co-cultured in the channel and on the porous membrane, respectively to construct in vitro blood-air barriers. The channel dimensions in the device can be adjusted to simulate in vivo dynamic flow conditions.
